# Psoriasis, Is It a Microdamage of Our “Sixth Sense”? A Neurocentric View

**DOI:** 10.3390/ijms231911940

**Published:** 2022-10-08

**Authors:** Balázs Sonkodi

**Affiliations:** Department of Health Sciences and Sport Medicine, Hungarian University of Sports Sciences, 1123 Budapest, Hungary; bsonkodi@gmail.com

**Keywords:** psoriasis, Piezo2 channelopathy, compression axonopathy, ganglionopathy, non-contact injury, HSP70, interleukin-6, TLR-4, Wnt signaling, lactate

## Abstract

Psoriasis is considered a multifactorial and heterogeneous systemic disease with many underlying pathologic mechanisms having been elucidated; however, the pathomechanism is far from entirely known. This opinion article will demonstrate the potential relevance of the somatosensory Piezo2 microinjury-induced quad-phasic non-contact injury model in psoriasis through a multidisciplinary approach. The primary injury is suggested to be on the Piezo2-containing somatosensory afferent terminals in the Merkel cell–neurite complex, with the concomitant impairment of glutamate vesicular release machinery in Merkel cells. Part of the theory is that the Merkel cell–neurite complex contributes to proprioception; hence, to the stretch of the skin. Piezo2 channelopathy could result in the imbalanced control of Piezo1 on keratinocytes in a clustered manner, leading to dysregulated keratinocyte proliferation and differentiation. Furthermore, the author proposes the role of mtHsp70 leakage from damaged mitochondria through somatosensory terminals in the initiation of autoimmune and autoinflammatory processes in psoriasis. The secondary phase is harsher epidermal tissue damage due to the primary impaired proprioception. The third injury phase refers to re-injury and sensitization with the derailment of healing to a state when part of the wound healing is permanently kept alive due to genetical predisposition and environmental risk factors. Finally, the quadric damage phase is associated with the aging process and associated inflammaging. In summary, this opinion piece postulates that the primary microinjury of our “sixth sense”, or the Piezo2 channelopathy of the somatosensory terminals contributing to proprioception, could be the principal gateway to pathology due to the encroachment of our preprogrammed genetic encoding.

## 1. Introduction

Psoriasis is considered to be a chronic multifactorial and heterogeneous systemic disease with a number of underlying pathologic mechanisms having been revealed; however, the pathomechanism is far from entirely known. The clinical picture is associated with chronic immune-mediated inflammation with remissions, dysregulated keratinocyte proliferation, and differentiation.

Epidemiological studies have estimated the prevalence of psoriasis to 3% of the adult U.S. population; this has not changed since 2003 [1]. This also means that psoriasis is one of the most common immune-mediated disease among U.S. adults [1]. More than 80 psoriasis susceptibility regions have been identified based on genome-wide association studies [2], such as the genes related to Th17 cell activation [3]. Non-genetic, environmental risk factors in psoriasis entail drugs, smoking, drinking, diet, infection, and mental stress [4].

Emerging research has highlighted the neurogenic inflammation aspect of psoriasis. Some studies have even reported the dysregulated activation of nociceptive neurons [5], not to mention the phenomenon that remission or improvement could occur due to the absence of neural contributions [6]. Furthermore, many studies have demonstrated that the innervation of lesioned psoriatic skin is greater than non-psoriatic skin; this applies to the number of nerves, the density, the total length, and the proportion of these nerve penetrating into the epidermis [5,7,8,9,10,11,12]. However, the author proposes that the primary or critical microinjury is at the somatosensory terminals due to mechano-energetic impairment; therefore, the secondary or tertiary dysregulated nociceptive activity is the direct result of primary damage, in addition to genetical predisposition and environmental risk factors. The neuro-energetic extension of homeostasis is allostasis, meaning the maintenance of stability in an energy-demanding perturbed environment, such as in severe physical or mechanical challenges [13]. Accordingly, the acute or chronic state of allostatic stress could also have relevance in the initiation and progression of psoriasis. The author of this manuscript suggests that the somatosensory afferent terminals of the Merkel cell–neurite complex could be microdamaged by repetitive forced longitudinal stretch under an acute or chronic stress response time window, as could be the case in delayed-onset muscle soreness (DOMS) [14]. Correspondingly, it is proposed that Piezo2 ion channels at the Merkel cell–neurite complex could be subjected to pathological mechano-energetic microdamage, and as a result could become “leaky” during allostatic stress, such as in DOMS [15,16,17]. Notably, the proposed types of sensory terminal lesions, named terminal arbor degeneration (TAD), evolve after a threshold-driven, dose-limiting manner in an acute and chronic fashion, not associated with Wallerian-like axonal degeneration [16,18].

Hereafter, the current opinion piece will demonstrate the relevance of the somatosensory terminal Piezo2 microinjury-induced quad-phasic non-contact injury model in psoriasis with a multidisciplinary approach. Notably, the proposed injury mechanism concerns when the pathophysiology is initiated, what the critical path is in the initiating phase of pathomechanism, and what is the longitudinal neural consequence of the primary damage; the intention is certainly not to refute any prior findings or theories.

## 2. Piezo Ion Channels

In order to reveal the proposed neural microinjury mechanism of psoriasis, the suggested microinjury site, namely, the Piezo channels should be introduced. Piezo proteins are evolutionarily conserved, giant force-gated excitatory mechanosensitive and nonselective ion channels with numerous transmembrane segments [19]. Indeed, they are the largest pore-forming transmembrane ion channel proteins explored to date [20]. Their role is essential in the mechanotransduction of life-sustaining signals, such as touch sensation, proprioception, and cardiovascular regulation [21]. Two types of Piezo proteins are found in humans, namely, Piezo1 and Piezo2, and both channels have interesting structural properties, such as their propeller blades and similar conformations [22]. Notably, both Piezo ion channels have their own distinguished mechanically activated currents in overexpressed states [23]. Nevertheless, questions remain in reference to topology and functions, such as pore formation, mechanical force detection, and gating [19].

Mechanotransduction conveyed by Piezo1 channels has an important role in the homeostasis maintenance of peripheral tissues, such as in skin [24], cartilage [25], blood pressure regulation [26], and urinary osmolarity [27]. Moreover, Piezo1 channels play a role in cell alignment due to their shear stress sensor capability [28,29]; this signaling could be relevant in the loading of tissues, such as muscles, bones, joints, and even skin [24], not to mention their suggested role in remodeling [30]. On the other hand, Piezo2 ion channels contribute to the homeostasis maintenance of somatosensory neurons [28,31,32].

In summary, the Piezo1 channels of peripheral tissues serve the purpose of being cellular mechanoceptors, but they could be neuromodulators as well through crosstalk with Piezo2 ion channels at the somatosensory terminals. Moreover, Piezo2 channels are suggested to have a principal function of being homeostatic gatekeepers of the central nervous system (CNS) [33]. The author proposes that the functionality of this gatekeeper function of Piezo2 could fail under allostatic stress due to mechano-energetic impairment.

Notably, Piezo2 was found to be the principal mechanotransduction channel for proprioception [34]. Proprioception was formerly known as “sixth sense” by a distinguished Scottish anatomist, physiologist, neurologist and surgeon, Sir Charles Bell, in 1830 [35]. We could define proprioception as our sense of detecting movement, action, and location, and thus, the awareness of the location of our limbs and body. It is controversial whether skin could contribute to the positional sensing of proprioception [36]. Noticeably, studies are emerging in support of the skin’s contribution to proprioception; more specifically, the contribution of sensory afferents of Merkel cell–neurite complexes [15,37].

## 3. Quad-Phasic Non-Contact Injury Model

Recently, it was hypothesized that unaccustomed or strenuous repetitive forced lengthening contractions under a cognitive demand-derived acute stress response time window could cause proprioceptive sensory nerve terminal microdamage in muscle spindles of striated muscles, leading to the primary injury phase of DOMS [14]. Notably, this primary injury is proposed to be a transient Piezo2 channelopathy in association with impaired glutamate vesicular release, and that it is pain-free [15]. The result of this somatosensory microdamage is impaired proprioception [18] and could be followed by secondary harsher tissue damage in DOMS, associated with the clinical picture of acute compression axonopathy [14]. Indeed, recent studies support this theory that mechanical allodynia is Piezo2-dependent after chronic nerve compression injury [38]. Proske and Gandevia suggested that damaging forced lengthening contractions are to blame for impaired proprioception [39]. Notably, research is also emerging that impaired proprioception is part of the clinical picture of psoriatic arthritis [40,41,42], not to mention the accumulating evidence on the involvement of neurogenic mechanisms in the disease pathomechanism of psoriasis [43,44]. Furthermore, the existence of an underlying systemic silent pathophysiology is telling; new psoriatic lesions could evolve due to mechanical injury on previously uninvolved [45], already dysregulated skin areas [46]. This phenomenon, named after the German dermatologist Heinrich Koebner, is apparent in many other cutaneous diseases [47], which could suggest that the implied primary gateway to pathophysiology enjoys some kind of principality, as claimed by Sonkodi et al. [14,15]. Fernández-Trillo et al. also proposed that Piezo2-containing neurons are key and may have different genetic signatures [48]. Indeed, recent papers addressing Piezo2 microinjury suggest that it could be a principal gateway to pathology [14,15,18,30,33,49]. The tertiary injury phase in DOMS is called the repeated bout effect, and is basically the re-injury of the initiating primary sensory terminal microdamage by the same repetitive forced lengthening contractions under an acute stress response [15]. Repetitive re-injury of these non-contact injuries could evolve into chronic conditions or to ganglionopathy associated with genetic predisposition or environmental risk factors, as suggested in osteoarthritis (OA), post-orgasmic illness syndrome (POIS), dry eye disease (DED), and osteoporosis [15,30,33,49]. This concept has already been hypothesized, in the association of repetitive microdamage and mechanical stress with the triggering of lesions in psoriatic arthritis [50,51,52,53]. Finally, the quadric phase of the injury mechanism is suggested to be the aging process and the associated inflammaging (see Table 1) [33,49,54].

### 3.1. Important Underlying Factors

#### 3.1.1. Repetitive Forced Longitudinal Stretch

An important clinical observation is that psoriasis frequently manifests in locations where the skin is rubbed and undergoes strong repetitive mechanical, such as the elbows, knees, and the scalp [55]; indeed, shear stress could be relevant in the proposed somatosensory microdamage [14,30,33,49]. In addition, it has been suggested that mechanical stretch might be a factor in the pathogenesis of psoriasis [55,56]. Accordingly, research has demonstrated that mechanical stretch at higher intensities leads to significant epidermal hyperproliferation and cytokine upregulation in psoriatic skin [55].

Repetitive mechanical stretch excites or hyperexcites multiple different types of skin cells, but more importantly, even somatosensory neurons as well. The Cox2–PGE2 pathway plays a role in this neural excitatory process, possibly leading to hyperexcitation as in other tissues, e.g., muscles and bones [14,30]. Furthermore, the pivotal roles of bradykinin and nerve growth factor (NGF) come into play under hyperexcitation [14,30,57]. Indeed, keratinocytes release NGF as target cells for sensory neurons and sympathetic neurons, and these neurons express their NGF receptors at the distal axons [58,59]. NGF is released when ischemia or nerve injury occur [60,61]. Notably, at this stage, we could talk only about excitation/hyperexcitation/pathological hyperexcitation, and transient pathological hyperexcitation should not be interchanged with the term sensitization, which is suggested to be a chronic micro-injured state of Piezo2. Bradykinin enhances Piezo2 currents, whereas NGF plays a role in the noxious stimulation of Piezo2 [62,63].

Moreover, it has been shown that keratinocytes express high levels of NGF in both lesional and nonlesional psoriatic tissue [64]. Additionally, the marked upregulation of NGF receptors could be observed in the cutaneous sensory terminals of psoriatic lesions in addition to the p75 neurotrophin receptor (p75NTR), tyrosine kinase A (TrkA), substance P, and calcitonin-gene-related protein (CGRP) [64]. Furthermore, the hallmark proliferation of sensory fibers could be observed in transplanted psoriatic plaques as opposed to the few nerves in transplanted normal human skin [64]. Notably, these hallmark peripheral findings of psoriasis may already be part of the sensitization mechanism of the disease, mentioned later, and could reflect that “part of wound healing kept alive permanently”, at least partially due to chronic Piezo2 channelopathy [33].

#### 3.1.2. Potential Role of The Stress Response

The aforementioned proprioceptive sensory terminal microinjury theory implied that the acute stress response is an important underlying factor in non-contact injuries [14,15,18,30]. Correspondingly, it has been observed that stress plays a role in the pathophysiology of psoriasis. Studies have often revealed that in addition to mechanical stress, emotional stress is an exacerbating factor in psoriasis [43]. Notably, melanocortin is a key regulator of the stress responses of skin. Moreover, the expression of genes in the melanocortin system and the melanogenesis enzymes are altered in psoriasis, i.e., the melanogenesis related genes are downregulated, whereas endogenous opioid system genes are upregulated, referring to the aforementioned dysregulated apthophysiology in psoriasis [65]. In addition, the stress response is dysregulated in psoriatic uninvolved skin, not to mention the underlying altered keratinocyte stress response [46]. The discordant autonomic response could be partially due to the impaired crosstalk between the autonomic nervous system and the micro-injured sensory Piezo2, as theorized by Sonkodi et al. [15].

Correspondingly, the author suggests that repetitive forced lengthening or longitudinal stretch under an acute and chronic stress response time window play a pivotal role in the neurogenic pathomechanism of psoriasis. It is important to highlight how somatosensory neuron terminals in the Merkel cell–neurite complex are impacted mechano-energetically by allostatic stress; the author suspects that repetitive forced longitudinal stretch damages these sensory neuron terminals, especially when the superposition of compression forces, including shear force, is factored, because forced lengthening contractions are damaged in DOMS.

#### 3.1.3. Terminal Arbor Degeneration-like Lesions Versus Wallerian Degeneration

It is an important distinction that the suggested critical and primary sensory terminal microinjuries are TAD-like lesions, as suggested by Bennet et al. and determined from paclitaxel-based chemotherapy [18,66]. These types of neuron terminal lesions do not come with classical Wallerian-like degeneration processes [66]; hence, the injury mechanisms and regeneration processes are different. The author translated this difference as somatosensory terminals contributing to proprioception which are constantly going through transient TAD-like lesions due to the concept that they are responsible for growth and remodeling [14,30,33,49]. This concept may arise from the Hilton law, which indirectly implies that bones, muscles, and skin can possibly grow concomitantly in a similar fashion under sensory guidance [14,67].

The author proposes that sensory nerve guidance in the Merkel cell–neurite complex has similar functional and essential roles in skin growth; it can be observed in bone growth and is suggested in muscle growth [14,67]. Correspondingly, remodeling in these tissues is proposed to be the revival of sensory-neuron-derived growth mechanisms under allostatic stress, as suggested in DOMS [14]. This is a transient process, such as in DOMS; however, repetitive re-injury with genetical predisposition and environmental risk factors could evolve into a chronic process, as suggested in psoriasis by the author.

Moreover, it is important to note that paclitaxel indeed potentiates Piezo2 currents in Merkel cells [68]. Hence, the paclitaxel-induced somatosensory hyperexcitation could be exacerbated, causing TAD-like lesions in somatosensory terminals in the Merkel cell–neurite complex. This microdamage could occur in acute and chronic fashion and in a dose-limiting manner [66]. This interpretation implies that these sensory afferent nerves have efferent functions on the periphery, not just afferent utilities. The author postulates that the efferent function not only relies on neuropeptides; tethering and Piezo currents could contribute to it as well. Furthermore, imbalanced subthreshold Piezo currents could be principally responsible for the dysregulated responses in psoriasis.

Finally, the reverse Koebner response should be mentioned; this is the disappearance of existing skin lesions due to trauma [69]. The author suggests that the reverse Koebner response could be explained by trauma-induced Wallerian degeneration of disrupted axons, such as those that had been previously exposed to TAD-like lesion at the terminal end.

### 3.2. Primary Injury Phase—Piezo 2 Channelopathy

Merkel cell–neurite complexes can be found in human fingertips, touch domes in the skin, and whisker hair follicles in high density [68,70,71]. These compartments are in close anatomical locations where psoriasis could evolve [55]. The Merkel cell–neurite complex consists of Merkel cells and Aβ somatosensory afferent endings to comprise a synaptic-like compartment [68,70]. Its complex functions cover the transduction of touch, social interactions, and tactile discrimination [68,72]. Its role in social interactions could be telling about the link why emotional stress exacerbates psoriasis, as mentioned previously [43].

Chang et al. reported that Piezo2 currents in Merkel cells are significantly enhanced by the microtubule stabilizer paclitaxel, as mentioned above, but reduced by the microtubule destabilizer vincristine [68]. These findings are in line with the earlier acute compression axonopathy theory of DOMS, developed by Sonkodi et al., who used the analogy from paclitaxel-based therapy in order to postulate that primary and critical injury in DOMS could be due to a mechano-energetic lesion of the proprioceptive terminals in the muscle spindles in a threshold-driven, dose-limiting manner [14,18]. Sonkodi et al. even put forward that the transient channelopathy of Piezo2 could be the critical cause of DOMS [15]. The inactivation of Piezo2 due to hyperexcitation occurs in homeostasis [73,74]. However, the microinjury of Piezo2 breaches the limits of the channel’s functionality under an over-reaching response or allostasis [15,54]. The proposed result of this mechano-energetic impairment is the “leakiness” of these channels to imbalanced subthreshold Ca^2+^ currents, and even to glutamate [15,33,49,54]. The mechanism suggested above is one principal gateway to pathology and to sensitization.

Recently, it has been highlighted that the periosteum is a similar compartment, as are muscle spindles in the striated muscles when it comes to sensory innervation [14]. Similar analogies could be drawn between the Merkel cell–neurite complex and the muscle spindle [75]. For example, an analogous synaptic-like vesicular release machinery has been identified in Merkel cells, as in muscle spindles [75]. Recently, it was demonstrated by Than et al. that this machinery in the muscle spindles is glutamatergic autoexcitation-based, providing the static-phase firing sensory encoding, as proposed previously by Bewick et al. [73,76]. Even more recently, it has been hypothesized that the mechano-energetic impairment of this glutamate vesicular release system and the transient proprioceptive Piezo2 channelopathy in the muscle spindle could lead to DOMS [15,16,54]. The suggested result of this impairment of glutamate vesicular release is the switch of static-phase firing encoding of the stretch reflex from impaired Type Ia sensory fibers to compensatory Type II fibers [16]. Notably, again, this type of microinjury not only exists on an acute basis, but in a chronic fashion as well [18,66]. Accordingly, it has been theorized that this type of chronic Piezo2 channelopathy on primary somatosensory neuron terminals could also lead to, e.g., non-contact anterior cruciate ligament injury, POIS, DED, and osteoporosis [15,30,33,49].

Casting out Merkel cells, or even more precisely, Piezo2 in Merkel cells, eliminates the late-phase firing of the touch response [32,75,77,78]. The remnant beginning phase firing is due to mechanically sensitive large Aβ fibers that are low-threshold mechanoreceptors and also express Piezo2 [31,75]. Importantly, blocking the vesicle release system of Merkel cells showed that the neurotransmitter release of Merkel cells is also essential for the static-phase firing of Aβ low-threshold mechanoreceptors [75,79]. However, there is still controversy about the type of neurotransmitter release that mediates prolonged static-phase firing in Merkel cell afferents [75,79]. The author emphasizes the importance of glutamate release, as demonstrated by Higashikwa et al. [75,80]. Glutamate is the likely candidate due to its contribution to neural injury signaling and pain sensation. Impairment of this vesicular release machinery could be one reason why glutamate is significantly elevated in the serum of patients with plaque psoriasis [81]. Accordingly, the author theorizes that the stress-induced impairment of prolonged static-phase firing and the Piezo2 channelopathy could lead to lost position control of cell orientation in the epidermis, as was hypothesized in the primary injury phase of DOMS [14,15,16,17]. Piezo2 channelopathy also means the impaired cross-talking between Piezo2 and Peizo1, as was suggested in DED [33]. Furthermore, the author suggests the essentiality of this intact Piezo cross-talking. The lack of intact Piezo crosstalk could be the reason why wound healing takes longer with Piezo1 in keratinocytes then without it [24]. Indeed, keratinocyte Piezo1 is essential for sensory afferent firing [82], but this communication is suspected to be two-sided [33]. The question was addressed by Holt et al., determining whether inhibiting Piezo1 in order to speed up wound healing would cause any detrimental effect [24].

Overall, acute and chronic somatosensory Piezo2 channelopathy could play an essential role in psoriasis lesion initiation and sustainment with genetic predisposition and environmental risk factors in the background. Chronic Piezo2 channelopathy will result in sensitization which is associated with low-grade neuroinflammation and “part of wound healing kept alive permanently”, instead of transiently, as was suggested in DED [33]. Indeed, loss-of-function mutations in Piezo2 cause losses of pain and sensitization as well [83], again highlighting the principality of these ion channels. Correspondingly, Sonkodi et al. emphasized that the static-phase firing encoding of the stretch reflex in association with Piezo2 channelopathy could cause ultradamage to our most profound life-sustaining preprogrammed genetic encoding [15]. Notably, they did not exclude the opportunity that Piezo2 of the penile skin could contribute to the proposed muscle-spindle-based Piezo2 primary damage in POIS [15]. Moreover, erections could be one example when skin could contribute extensively to position sense. However, these dermal somatosensory nerve terminals contributing to proprioception could be microdamaged due superposition of compression forces by repetitive forced lengthening contractions, thus providing the base for genital psoriasis in the presence of genetical predisposition and environmental risk factors.

In summary, impaired Piezo2–Piezo1 communication due to the chronically impaired functionality of microdamaged Piezo2 somatosensory terminals in Merkel cell–neurite complexes in psoriasis may ignite the uncontrolled proliferation and differentiation of sensitized keratinocytes, explained later. Hence, the microinjury to Piezo2 may represent one principal gateway to pathophysiology with longitudinal consequences.

### 3.3. Secondary Injury Phase—Compression Axonopathy

The dichotomous nature of the non-contact injury mechanism of DOMS has been observed [14,84,85]. The result of the primary pain-free proprioceptive terminal Piezo2 channelopathy and disabling the glutamate vesicular release is impaired proprioception and consequent secondary harsher tissue damage in DOMS [15,16,18]. The acute compression axonopathy theory of DOMS hypothesized a compensatory cross-talking of Type Ia and Type II (Aβ fiber) proprioceptive sensory neurons within the intrafusal space in the primary damage phase, followed by cooperative crosstalk with the interlinked Type III (Aδ fiber) and C-fiber neurons in the extrafusal space of the secondary damage phase after breakdown of the selective barrier of the muscle spindle [14,18]. The cross-talking of Type III and C fibers is due to the secondary harsher tissue damage, namely, in the muscle, connective tissue, or extracellular matrix (ECM). One proposed basis for this cross-talking is Piezo2-channel-based [15,17,33,54]. The chemical and enzymatic destruction of the ECM impairs Piezo2 mechanogating [22]; as a result, it could provide one way for the temporal summation of pain by nociceptive C-fibers in DOMS [14,17,86].

A fairly similar pathomechanistic cross-talking of sensory fibers in psoriasis is suggested by the author. Accordingly, Merkel cells are functionally analogous with the intrafusal Type Ia fibers; Aβ afferents of the Merkel cell–neurite complexes are analogous with intrafusal Type II fibers; and the Aδ fibers are analogous with axtrafusal Type III fibers, not to mention the analogous nociceptive C-fibers. Keratinocytes are peripheral cells, such as muscle cells in DOMS, which excite or hyperexcite Merkel cell–neurite complexes as neuromodulators.

The author proposes that psoriatic skin loses elasticity acutely due to the primary damage phase at the initiation of the psoriatic process, and later evolves into a chronic state of mechanical locking with low hysteresis [87]. Notably, protein filaments, synthesized by sensory neurons of skin, tether mechanosensitive ion channels in sensory neurons to the ECM [88]. These protein-sensitive links are responsible for the gating of cutaneous mechanoreceptors in order to transduce mechanical stimuli [88]. The destruction of these tethers ceases mechanosensitive currents and the transduction of mechanical stimuli without interfering with the electrical excitability of these sensory afferents [88]. Moreover, the Piezo1 ion channels of keratinocytes are critical for these Aδ sensory afferents’ firing and behavior responses to innocuous and noxious mechanical stimulation [82]. This further provides evidence of the importance of cross-talking between Piezo1 and Piezo2 ion channels. As mentioned previously, chemical and enzymatic destruction of the ECM impairs Piezo2 mechanogating [22]. Recent findings even demonstrated that the NGF–TrkA–Piezo2 signaling axis plays a role in noxious mechanical stimulation of Aδ sensory afferent neurons in bones [63]. Correspondingly, Piezo2 could exert a similar noxious mechanical stimulatory role on Aδ sensory fibers in the epidermis under repetitive forced longitudinal stretched environments, because there is a marked upregulation of NGF and TrkA in the terminals of sensory afferents of psoriatic lesions [64]. In line with the suggested secondary injury phase, there are increased expression levels of HPSE2, SYND1, MMP9, and TIMP2 in the ECM of psoriatic patient, even in the absence of psoriatic plaques, suggesting the involvement of these molecules in the initiation of the sensitization and psoriatic processes [89,90,91]. Notably, metalloproteinases such as MMP9 have an essential role in remodeling by impacting immune cell activation and inflammatory responses [89]. The elevated MMP9 level is even suspected to be one reason behind the ECM rupture in psoriatic lesions due to the inhibition of remodeling [89]. This alteration of the ECM in psoriasis is a significant distinction, because the ECM is an important regulatory space for proliferation, differentiation, metabolism, and remodeling, not to mention that the ECM is a key player of the healing process.

Importantly, painless primary microlesions do not need to correlate with the exact location of psoriatic lesions, as in the case of DOMS [14], because the secondary harsher tissue injury is due to the separate superposition of compression and lengthening longitudinal stretching forces, including shear stress, which eventually destroy the ECM enzymatically and, in the case of re-injury and genetical predisposition and/or environmental risk factors, this could initiate the psoriatic process.

This manuscript only proposes the Piezo2 channelopathy as the critical gateway to pathophysiology, and certainly does not exclude the possible involvement of other ion channels and receptors, although only in secondary or tertiary fashion. Indeed, the expression of TRP channels, TRPV1 and TRPA1, shows marked elevation in psoriatic skin due to the tertiary sensitization process mentioned later [5,92,93]. Accordingly, the dysregulated activation of nociceptive neurons in psoriasis [5] is suggested to only evolve in the secondary and tertiary injury phase as well.

### 3.4. Tertiary Injury Phase—Ganglionopathy

The repeated bout effect of DOMS shows that non-contact injuries have longitudinal dimensions, because the same severe bout of eccentric exercise is remembered for 6 months, resulting in a reduced DOMS feeling [94]. Indeed, proprioception is closely associated with memory and learning; therefore, microinjury of these somatosensory terminal Piezo2 channels is proposed to impact learning and memory in the CNS [18]. Sonkodi et al. proposed that the tertiary non-contact injury phase is the consequence of the repeated transient re-injuries of Piezo2 channels leading to chronic Piezo2 channelopathy with learning and memory consequences [15,30]. Therefore, overloading earlier transient Piezo2 channelopathies could lead to vulnerability to repeated damage, in association with neuroinflammation and potential genetical predisposition and/or environmental risk factors in the background, which could induce the tertiary damage phase.

Healing of the secondary injury phase is a transient compression axonopathy. However, repeated injury and genetical predisposition and/or environmental risk factors could derail this transient process into a chronic state of “part of wound healing kept alive permanently” [33,49]. Notably, the repetitive painless primary injury phase is theorized to bypass the secondary damage phase and evolve into the tertiary injury phase in certain cases, as is suggested in POIS [15]. This phenomenon further highlights the importance of the silent primary microdamage phase.

One characteristic feature of psoriasis is the dysregulated proliferation of activated keratinocytes [95]. The author translates this phenomenon as the result of the following processes.

#### 3.4.1. Wnt Signaling Pathway

It has been theorized that chronic Piezo2 channelopathy could shift the canonical Wnt signaling pathway toward noncanonical signaling pathway [49]. Wnt signaling pathway is transduced through cell surface receptors into cells by proteins and it has an essential role in tissue homeostasis [96]. These proteins are highly conserved evolutionarily [97], similarly to Piezo ion channels [19]. There are three Wnt signaling pathways: the canonical Wnt pathway and the noncanonical Wnt pathway/calcium pathway. The noncanonical Wnt pathway is responsible for cell polarity and migration, whereas the noncanonical Wnt/calcium pathways are responsible for Ca^2+^ and apoptosis regulation within the cell [98]. Indeed, the above shift from the canonical Wnt signaling pathway toward the noncanonical signaling pathway could be observed in psoriasis driven by Wnt-5, associated with the impaired homeostatic inhibition of Wnt signaling [99,100]. This Wnt signaling inhibition is due to WIF-1 and Dickkopf [99], but at the early stage, the role of interleukin-6 (IL-6) should not be excluded [49]. Indeed, IL-6 could negatively interact with the Wnt signaling pathway in rheumatoid arthritis [101]. Moreover, IL-6 is abundantly expressed in psoriatic skin and enhances the proliferation of keratinocytes [102]. The result of Wnt signal inhibition could be detrimental on Piezo2 due to lost stimulator effects on phosphatidylinositol [4,5] bis-phosphate (PIP2) formation [54]. Notably, Piezo2 mechanotransduction is also under the control of PIP2 in peripheral sensory neurons [103]. Therefore, this Wnt signaling pathway shift could lead to the microdamaged functionality of Piezo2 channels, not to mention the imbalance subthreshold Ca^2+^ currents which induce unfinished healing processes, as suggested by Sonkodi et al. [33]. Furthermore, it is agreed that canonical Wnt signaling is an anti-inflammatory mechanism, whereas the noncanonical pathway is pro-inflammatory when it comes to neuroinflammation [104,105,106]. The pro-inflammatory fashion of the noncanonical pathway in neuroinflammation is partially due to the stimulation of the nuclear factor-kappa B (NF-κB) pathway [105]. Indeed, the NF-κB pathway is activated in DOMS, and in psoriasis it is even proven to be essential in the pathomechanism [107,108].

#### 3.4.2. NK and NKT Cell Activity and Autoinflammation

Noncanonical Wnt receptor Ryk functionally regulates natural killer (NK) cell development temporally [98]. Indeed, inflammatory infiltrates of psoriatic skin contain CD^3−^/CD^56+^ NK cells [109]. These NK cells, stimulated by interleukin-2, could activate keratinocytes [109].

However, what could be even more relevant in reference to the proposed primary Piezo2 microinjury is the finding that CD^3+^/CD^56+^ natural killer T (NKT) cells are significantly decreased in the peripheral blood of psoriatic patients [110], as in rheumatoid arthritis [111]. Notably, recently published observations have demonstrated elevated CD^3+^/CD^56+^ NKT cells in DOMS [112]. Sonkodi et al. attributed this effect to the heat shock protein 70 (Hsp70) activated Toll-like receptor 4 (TLR4)/IL-6 (IL-6)/tumor necrosis factor alpha (TNF-α) pathway, based on the work of Dos Santos et al. [112,113]. The upregulation of Piezo2 and IL-6 seems to be paralleled in the dorsal root ganglion (DRG) of chronic neural constriction injury [114]. The author translates this phenomenon as one consequence of the chronic Piezo2 channelopathy being this upregulation and ganglionopathy. Furthermore, transient Piezo2 channelopathy could elevate NKT cells, but chronic Piezo2 channelopathy could deplete circulating NKT cells in the peripheral blood. Moreover, the depletion of circulating NKT cells could be one explanation for the dysregulation of invariant NKT cells in psoriasis downstream, leading to increased relative frequencies of iNKT2 and iNKT17 cells, and decreased relative frequencies of total and CD69^+^ iNKT cells [115,116]. IL-6 could play a role in the inhibition of Wnt signaling pathway, as proposed above, providing one feasible explanation why IL-6 expression is elevated in psoriatic skin and stimulates keratinocyte proliferation [102]. Notably, IL-6 could cross selective barriers [117], and this trans-barrier signaling could be relevant in the crossing of the selective barrier of the muscle spindle in DOMS and in psoriasis disease progression.

NKT cells are both innate and adaptive immune cells, but belong to the innate arm of the immune system [118]. They are activated as the first line of the immune response and are capable of activating other cells downstream without formulating immune memory, but they also have a role in the initiation and progression of autoimmune diseases [118]. Another important characteristic of NKT cells is that they can recognize exogeneous and endogenous glycolipids, leading to the activation of these cells during bacterial infection [119]. This feature is important, because the result of pathological hyperexcitation is the proposed mechano-energetic microinjury-derived “leakiness”, which might provide access to NKT cells to recognize the degradational debris of dysfunctional mitochondria. Notably, mitochondria evolved from bacteria [120], and research is emerging which shows that the “self-eating” of damaged mitochondria is not always completely fulfilled by neurons leading to their release through axon terminals [121]. Interestingly, it has been hypothesized that psoriasis is the cross-activation of an autoimmune process between structural proteins of keratinocytes and streptococcal antigens [119]. Overall, the author proposes that mitochondria debris from leaky somatosensory terminals of the Merkel cell–neurite complex could play a role in the initiation of the autoimmune and autoinflammatory processes in psoriasis.

#### 3.4.3. HSP 70

Both the innate and adaptive immune responses are involved in a dysregulated way in the pathogenesis of psoriasis [119]. The release of endogenous stimuli, named damage-associated molecular patterns (DAMPs) [122,123], could activate pattern recognition receptors (PRRs) which are central players of sterile inflammatory processes [122,124]. DAMPs are often shed into the cytoplasm as a result of CNS injury [122]. The resultant chronic activation of these receptors could lead to inflammatory diseases [122]. Heat shock proteins, such as Hsp70, are examples for important DAMPs in psoriasis [125], as in DOMS [113]. Notably, the association between ion channel expression and the activated innate immune system and inflammatory response in the pathogenesis of several diseases has been observed [126]. TLRs are highly relevant PRRs in the nervous system, contributing to the initial immune response and connecting the first-line unspecific defense with secondary adaptive immunity [127]. Notably, external TLRs detect bacterial proteins [106], and possibly mitochondrial proteins as well. The activation of these receptors induces the downstream release of NF-κB [128,129], and NF-κB is an essential player in the pathomechanism of psoriasis and DOMS, as mentioned earlier [107,108].

Hsp70 is a molecular chaperone, but also plays a role in polypeptide folding, protein transport, and prevents protein aggregation [130]. These heat shock proteins could construct membrane rafts in association with membrane lipids, such as cholesterol and sphingolipids [131]. Furthermore they could exert so-called “moonlighting” activities by preserving the structural and functional stability of membranes under stress, or even more importantly, under allostatic conditions [132]. Hsp70 is known to bind to bis-phosphate in a pH-dependent manner of the inner membrane of vesicles: it is cytoprotective [130]. However, the prevention of this interaction between Hsp70 and bis-phosphate leads to lost cytoprotective impact [133], and sensitivity to vesicular damage due to oxidative stress further downstream [130]. The author proposes an analogous membrane instability mechanism that leads to the impairment of glutamate vesicular release and Piezo2 channelopathy in Merkel cells, as was proposed in DOMS [15,16,54]. Moreover, Wnt signal inhibition could be detrimental on Piezo2 due to the lost formation of a bis-phosphate, called PIP2 [54]. In addition, Piezo channels have a role in the control of endosome trafficking [134]. Moreover, the excitation of Piezo channels locally depletes membrane cholesterol [135,136]; however, cholesterol is also needed for membrane raft formation with Hsp70 [130,131].

Hsp70-like protein has been shown to excessively activate dendritic cells in the form of exosomes in the initial stage of psoriasis [137]. These activated dendritic cells release inflammatory cytokines, such as TNF-α, IFN-γ, and interleukin-17 (IL-17), and in return, these cytokines promote keratinocyte proliferation and psoriasis progression [138,139,140,141] with the involvement of the TLR4/NF-κB signaling pathway [142,143]. Promising findings of a recent study demonstrated that Yangxue Jiedu Soup significantly decreased the secretion of Hsp70-containing exosomes in the plasma of psoriasis model mice by downregulating the production of TNF-α, IL-6, IL-1β, IFN-γ, IL-17, and IL-23 [138]. The author proposes that in this inflammatory environment, even mitochondrial Hsp70 (mtHsp70) could be released due to the TAD-like mechanoenergetic lesion and the incomplete self-eating of dysfunctional-mitochondria-derived debris is shed through the “leaky” somatosensory terminals of the Merkel cell–neurite complex. Indeed, mtHsp70 has a known role in human diseases and in senescence [144,145].

Another telling finding could be that CD1d-restricted CD161^+^ NKT cells could release instantly IFN-γ if they encounter the glycolipid antigen presented by CD1d on keratinocytes in psoriasis [146]. This finding could be an important link in the initiation of autoinflammation and autoimmune processes, and the cross-talking between the adaptive and the innate immune system in psoriasis. Moreover, this important finding is in addition to the pre-psoriatic skin of psoriatic patients having substantially more CD161^+^ NKT cells compared with normal skin [147], suggesting possible reasons that could lead to the proposed silent NKT cell depletion downstream in psoriasis pathophysiology.

Overall, the cross-activation of an autoimmune process between psoriatic skin cells, such as keratinocytes, structural Hsp70, and mtHsp70 of somatosensory neurons of the Merkel cell–neurite complex, could be an important link in the autoinflammatory and autoimmune pathomechanism of psoriasis with NKT cell and TLR4/NF-κB signaling pathway involvement.

#### 3.4.4. Innate Immune System

The concomitant activation of keratinocytes by mechanical stretch could produce chemokines, and as a result, they could direct neutrophil and T cell migration [148,149]. Furthermore, they could also activate the innate immune system by expressing and secreting antimicrobial peptides (AMPs) [55,150,151]. AMPs only have relevance in the secondary or tertiary damage phase in order to maintain skin barrier homeostasis, and could be involved in immune dysregulation under pathological conditions, thus providing crosstalk between the innate and adaptive immune system in psoriasis [152]. Moreover, activated keratinocytes could enhance the cytokine storm, which could promote Th17-mediated immune responses in psoriatic lesions [153].

#### 3.4.5. Lactic Acid and Glutamine

High lactic acid levels in psoriasis are reported with high glycolytic activity [154]. System metabolomics approaches also show increased glutamine levels in psoriasis [154]. Elevated lactate levels used to be considered the cause of DOMS, although this theory was later refuted [155]. One interesting new theory, however, suggests that the impairment of dysfunctional-mitochondria-induced glutamate vesicular release could be associated with malfunctions of the lactate shuttle machinery in DOMS [156].

Correspondingly, the author proposes that activated mesenchymal stem cells could increase glycolysis in psoriasis in order to produce more lactate, as astrocytes do in ischemic episodes of the CNS when lactate is preferentially used by neurons over glucose [157]. This increased metabolic demand could be another indirect indication of neural microdamage in psoriasis. Indeed, mesenchymal stem cells increase keratinocyte proliferation and glycolysis in psoriasis, in addition to reducing keratinocyte junctions [158]. Notably, elevated lactate levels are not preferential to NKT cell survival and proliferation, in contrast to glutamine [159]. Glutamine could not only be important for NKT cell attraction, but for fueling the glutamate vesicular release machinery of Merkel cells, as it has been theorized to be the case in the Type Ia proprioceptive terminals of the muscle spindles [156]. However, impairment of the glutamate vesicular release machinery could lead to elevated glutamine levels in psoriasis, as system metabolomics approaches show.

In summary, the author of this manuscript propose that chronic somatosensory Piezo2 channelopathy or the permanent unwanted leakiness of these ion channels could also be blamed for the sensitization process in psoriasis, similarly to in DED [33], osteoporosis [49], and OA [30,160], which could be translated as low-grade neuroinflammation. and part of wound healing is kept alive chronically instead of transiently. One of the first-line consequences downstream could be the upregulation of Piezo2 channels in the DRG [114] of the affected somatosensory afferents of the Merkel cell–neurite complex and the resultant feed-forward sensitization of Piezo1 on activated keratinocytes, as could be observed in chondrocytes of OA [161]. Further discussion of the peripheral and central sensitization of psoriasis and their progressiveness is not the subject of this manuscript, only the aforementioned critical pathways. However, it is important to note again that there is no pain evolvement and sensitization in the absence of Piezo2 [83].

### 3.5. The Quadric Injury Phase—Aging-Associated Inflammaging

Aging-associated inflammaging is considered the quadric phase of this non-contact injury model [33,49]. However, the link between psoriasis and aging is more mysterious in contrast to other suspected chronic Piezo2 channelopathies, such as DED and osteoporosis. Notably, the life expectancy of psoriatic patients is significantly shorter than healthy controls [162,163]; some studies even estimate a lifespan reduction of 4 to 10 years [162,164,165]. Furthermore, comorbidities, such as cardiovascular diseases and diabetes, are also telling about this association [162,163]. Importantly, biological age differences could not be established within the lifetime of psoriatic patients, neither from involved and uninvolved psoriatic skin, nor from whole-blood testing [162,166].

However, the development of insomnia, depression, and anxiety in psoriatic patients hints at a pathway toward progressive neuroinflammation and neurodegenerative processes of the CNS [167,168]. Another interesting link toward neurodegeneration is the significantly increased incidence of Alzheimer’s disease among psoriatic patients [169]. Notably, crosstalk between the Wnt signaling pathway and TLR (including TLR4) is relevant in the neuroinflammatory process leading to Alzheimer’s disease [106].

Another interesting finding is that female patients present a significantly higher age difference than female healthy controls [162]. Borsky et al. translated their results as females reacting with stronger immune responses to infection and vaccination, with a higher resultant susceptibility to autoimmune diseases [162,170,171]. However, the author would like to propose the possible relevance of the NGF–TrkA–Piezo2 signaling axis in this sex difference [33].

Nevertheless, the aging mechanism of psoriatic patients and associated inflammaging are not the detailed subject of this paper.

## 4. Barrier Impairment in Psoriasis

Lost or impaired barrier function is relevant in the spreading of peripheral inflammation. Compartmentalization is an important protective feature of the nervous system as well, hence the impairment of these selective barriers of compartments could provide access to neuroinflammation progression.

Emerging research emphasizes the disruption of skin barrier function in psoriasis progression [172,173]. Correspondingly, Quiao et al. showed that barrier functions of the skin could indeed be impaired through dilator-induced mechanical stretch [55]. However, the progressive impairment of selective barriers does not stop at the compromised epidermal barrier because the progressive loss of selective barriers in the peripheral and central nervous system might not be regarded, especially if psoriasis is considered as a systemic disease.

According to the theory of Sonkodi, the propagation of neuroinflammation from the primary injury phase of non-contact injuries to the quadric phase implies the progressive disruption of these selective barriers in the peripheral and central nervous system as well [14,18]. Bradykinin is known to induce the breakdown of selective barriers in the CNS [174], even in transient fashion [175]. Correspondingly, it was theorized that it could increase the permeability of the selective barrier of the muscle spindle in DOMS [14]. However, bradykinin could play a role in the periphery as well in the increased epidermal barrier permeability. Notably, bradykinin 2 receptor (b2r) expression in several cell layers of psoriatic skin is increased in contrast to adjacent skin; however, the intensity of expression is diminished compared with adjacent skin [176]. Furthermore, b2r expression is negatively correlated with epidermal thickness [176]. These findings could be telling about the initial contribution of bradykinin in the psoriatic process. Critically, bradykinin is a key modulator of the hyperexcitation/pathological hyperexcitation mechanism, as noted previously, not to mention that the activation of b2r could increase the amplitude of Piezo2 currents and slow the inactivation of Piezo2 channels [62], possibly leading to the microinjury of Piezo2.

It is an important finding that the N-methyl-D-aspartate (NMDA)-type glutamate receptor plays a key role in the maintenance of cutaneous barrier homeostasis [177]. Notably, one suggested consequence of the impairment of the glutamate vesicular release is the activation of NMDA receptors due to glutamate spillover [15,16,18], possibly not only in the nervous system, but on the periphery as well. Therefore, glutamate also plays a key role in the epidermal hyperplasia of psoriasis induced by barrier disruption [177].

Finally, it is interesting to note that Piezo1 ion channels are responsible for cell orientation [28,29] and the regulation of osmolarity [178]; therefore, there is every ground to suspect that the Piezo1 of keratinocytes under the influence of impaired Piezo2 functionality could contribute essentially to dry skin in psoriasis beyond uncontrolled proliferation and differentiation. Indeed, trans-epidermal water loss is a well observed symptom in psoriatic patients and is associated with epidermal ceramide reduction [179]. The lack of ceramide could contribute to the instability of vesicular membranes under stress [130], which could lead to the impaired synaptic-like vesicular release of Merkel cells and eventually to the aforementioned Piezo2 channelopathy-induced impaired Piezo cross-talking that affects the Piezo1 of keratinocytes. An interesting novel finding is that membrane ruffling plays a role in the mechanosensation of extracellular fluid viscosity [180]. It is likely that Piezo channels deplete membrane cholesterol under pathological hyperexcitation, which could negatively impact the mechanosensation of membrane ruffling in association with Piezo channelopathy.

## 5. Ontogenetic Relevance

Psoriasis is primarily a human disease [181]. The author attributes this distinct disease condition to the erect and upright posture and bipedality of humans. As a result, proprioception has gained a more critical role to serve homeostasis of this sustained upright posture of humans. Therefore, the mitochondrial supply of somatosensory terminals contributing to proprioception is critical in an axon-length-limiting manner in order to fuel the neuroenergetic demand of proprioception, or more precisely, the Piezo system, especially in times of hyperexcitation under allostasis. This could be the reason why repetitive axial compression forces [30,49], repetitive forced lengthening contractions [14,15,16], and forced longitudinal stretching under allostatic conditions could be damaging to these somatosensory terminals contributing to proprioception, because they could deplete or disconnect the neuroenergetic supply of postural control in a noncontact or contact basis.

Hilton’s rule is suggested to lay the ground for the common sensory guiding of muscles, bones, and skin [14,15]. However, this sensory guidance not only serves the growth process and regeneration of muscles, bones, and skin, but also seems to play a critical role in remodeling [14,17,30].

Finally, the Piezo system in our muscles, bones, skins, eye, circulatory system, and nervous system could bring the tangibility of our “sixth sense”, or proprioception, into the limelight. However, the microdamaged functionality of these Piezo channels could occur in a transient fashion, serving the purpose of growth, regeneration, and remodeling, whereas in a chronic fashion this could evolve into disease conditions with thus far not entirely established pathomechanisms, such as psoriasis, OA [160], osteoporosis [49], POIS [15], DED [33], and many others.

## Figures and Tables

**Table 1:** The quad-phasic non-contact injury model adapted to psoriasis [33].

PIEZO2 MICROINJURY-INDUCED QUAD-PHASIC NON-CONTACT INJURY MODEL [33]				
**ENVIRONMENTAL FACTORS**		**PRIMARY INJURY PHASE**		**GENETICAL PREDISPOSITION**
	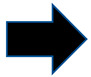	Repetitive forced longitudinal stretch	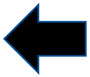	
		Fatigue-induced stress response		
	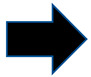	Stress-derived energy depletion of the mitochondria in the somatosensory terminal	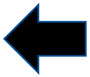	
		Mechano-energetic impairment of Piezo2		
		Painless compression Piezo2 channelopathy		
	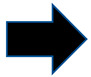	**SECONDARY INJURY PHASE**	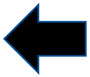	
		Harsher tissue damage due to impairment of Piezo2 with nociceptive fiber contribution		
		Painful compression axonopathy		
	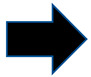	**TERTIARY INJURY PHASE**	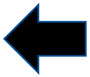	
		Re-injury and sensitization could evolve into chronic condition due to repetitive overloading		
	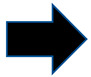	Chronic neuroinflammation or ganglionopathy Piezo2 upregulation in affected DRG ganglions Piezo1 upregulation on keratinocytes	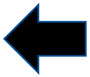	
		**QUADRIC INJURY PHASE**		
	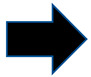	Aging or non-resolving neuroinflammation-induced Piezo2 microinjury or the augmentation of former channelopathy and ganglionopathy	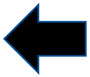	

## Data Availability

Not applicable.
